# Genome- and transcriptome-wide splicing associations with alcohol use disorder

**DOI:** 10.1038/s41598-023-30926-z

**Published:** 2023-03-09

**Authors:** Spencer B. Huggett, Ami S. Ikeda, Qingyue Yuan, Chelsie E. Benca-Bachman, Rohan H. C. Palmer

**Affiliations:** Behavioral Genetics of Addiction Laboratory, Department of Psychology, Emory University, 36 Eagle Row, Atlanta, GA 30322 USA

**Keywords:** Computational biology and bioinformatics, Genetics, Neuroscience, Medical research

## Abstract

Genetic mechanisms of alternative mRNA splicing have been shown in the brain for a variety of neuropsychiatric traits, but not substance use disorders. Our study utilized RNA-sequencing data on alcohol use disorder (AUD) in four brain regions (n = 56; ages 40–73; 100% ‘Caucasian’; PFC, NAc, BLA and CEA) and genome-wide association data on AUD (n = 435,563, ages 22–90; 100% European-American). Polygenic scores of AUD were associated with AUD-related alternative mRNA splicing in the brain. We identified 714 differentially spliced genes between AUD vs controls, which included both putative addiction genes and novel gene targets. We found 6463 splicing quantitative trait loci (sQTLs) that linked to the AUD differentially spliced genes. sQTLs were enriched in loose chromatin genomic regions and downstream gene targets. Additionally, the heritability of AUD was enriched for DNA variants in and around differentially spliced genes associated with AUD. Our study also performed splicing transcriptome-wide association studies (TWASs) of AUD and other drug use traits that unveiled specific genes for follow-up and splicing correlations across SUDs. Finally, we showed that differentially spliced genes between AUD vs control were also associated with primate models of chronic alcohol consumption in similar brain regions. Our study found substantial genetic contributions of alternative mRNA splicing in AUD.

## Introduction

Alternative mRNA splicing is the process where a single gene codes for multiple mRNA transcripts and consequently multiple proteins and gene isoforms with different structures and functions. Nearly 95% of human genes undergo alternative splicing^1^. Alternative mRNA splicing in the brain is a major contributor to both the genetic and neuromolecular pathology of psychiatric traits^2^. But researchers rarely investigate genome-wide or transcriptome-wide alternative mRNA splicing associations with substance use disorders.

Alcohol consumption induces abnormal alternative splicing events. That is, in tightly controlled experiments, alcohol use has shown to alter the combinations of protein coding (exons) and non-coding (introns) regions in particular transcripts as well as modify the expression of individual gene isoforms relative to naïve controls in brain tissues and cell types^3,4^. These data suggest that alcohol exposure might directly disrupt alternative mRNA splicing in specific genes. Post-mortem human brain studies identify alternative splicing associations with alcohol use disorder (AUD) highlighting specific gene isoforms among ion channels^5^ and neurotransmitter receptors^6^ as well as intracellular pathways and synaptic plasticity processes^7^. One study reported that AUD *causes* changes in mRNA splicing in the brain^7^, but did not explore the possibility of genetic influences, which would be inconsistent with causality.

Other research has identified links between genetic predispositions and RNA associations from human brain data on substance use disorders. Using a variety of different genomic methods, including gene-based associations, expression quantitative trait loci, and partitioned heritability, genetic associations with substance use disorders show overlap with differentially expressed genes and gene co-expression networks associated with these traits in post-mortem human brain data^8–10^. Thus, mRNA associations with AUD in human brain data—including alternative mRNA mis-splicing—could be due to drug exposure, genetic factors, or both.

Common forms of genetic variation, like single nucleotide polymorphisms (SNPs; individual DNA variants), account for a modest amount of variance in AUD^11,12^. AUD is polygenic and shares genetic risk with other substance use traits^13^. Outside of putative alcohol metabolism genes and neurotransmission genes, the biological basis of the genetic predisposition to AUD or problematic alcohol use remains elusive. One important mediator of genetic risk could be neuromolecular events as DNA variation has been shown to predict differentially expressed genes linked to AUD in addiction neurocircuitry^14^. How, or whether, alternative mRNA splicing mediates the genetic risk to AUD is unknown.

**Figure 1:**
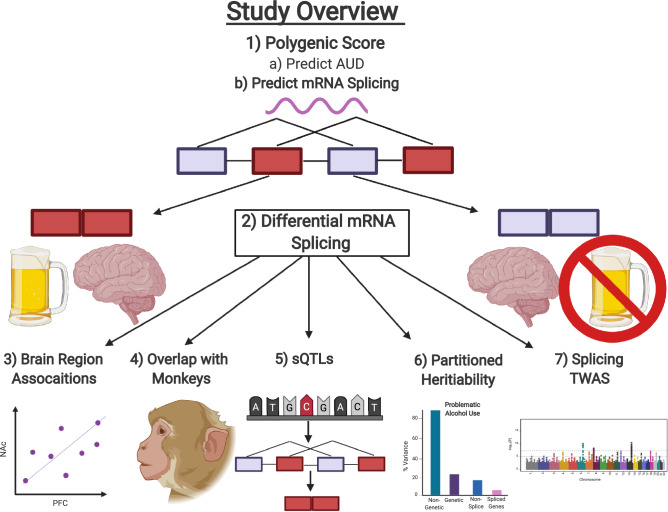
Schematic representation of our study. Created with BioRender.com.

Our exploratory study hypothesized that (1) polygenic scores of AUD would be higher in those with AUD than controls, (2) individual SNPs would be associated with abnormal alternative mRNA splicing in the brain, and (3) DNA variants around differentially spliced genes would contribute to the heritability of AUD. To test these hypotheses, our study used polygenic score analyses, splicing quantitative trait loci (sQTL) mapping (specific DNA variants that correlate with alternative mRNA splicing associated with a trait), partitioned heritability analyses, and estimated transcriptome-wide splicing associations from large-scale genome-wide association studies (GWASs). Using RNA-sequencing (RNA-seq) data from humans and primates, we also tested whether alternative mRNA splicing events were consistent across brain regions and whether differentially spliced genes linked with AUD overlapped with primate models of chronic alcohol use. For an overview of our study see Fig. 1.

## Materials and methods

### Samples

#### RNA-seq

We used the same publicly available data source of human post-mortem brain samples as Van Booven et al.^7^, which were collected from the New South Wales Brain Tissue Resource Center. Van Booven et al.^7^ also performed differential splicing, but they used different methods, included individuals from disparate ancestral backgrounds, and did not investigate genetic links with splicing. Using 56 Caucasian individuals^14^ (Supplementary Fig. S1), our study explored the possible genetic links of AUD-related splicing in the brain. Note, we derived genome-wide DNA variant information from RNA-seq brain data. Principal components analysis showed that individuals from the RNA-seq data were genetically homogenous, but did not cluster with the ancestral populations in 1000 Genomes reference samples—likely due to differences in data types (RNA-seq vs. GWAS). Still, systematic differences were observed between the ancestral clustering of RNA-seq data with genomic reference samples, which decreases the portability of polygenic score analyses. Of the human brain samples 23.22% were female and the average age was 57.34 (s.d. = 8.91, range = 40–73; see Table 1). AUD was defined as a diagnosis of either DSM-IV alcohol abuse (66.66% of AUD cases) or dependence (33.33% of AUD cases). Controls included social or non-drinkers that were not diagnosed with AUD and were well-matched on all covariates (see Supplementary Fig. S2). The most common cause of death was a cardiac complication (67.9% of all samples) followed by respiratory causes. Five individuals died of alcohol toxicity. Multiple brain regions were available for each individual and included: (1) superior pre-frontal cortex (PFC; PRJNA530758), (2) nucleus accumbens (NAc; PRJNA551775), (3) central nucleus of the amygdala (CEA; PRJNA551908) and (4) basolateral amygdala (BLA; PRJNA551909). Human brain samples were collected within three days of death (post-mortem interval range = 9–72 h, *M* = 32.81, s.d. = 13.75 h). For more information on the human RNA-seq data see Rao et al.^14^. Briefly, RNA was extracted via the Qiagen RNeasy kit. RNA was sequenced on the Illumina HiSeq 2000, which resulted in an average of 91,252,228 million paired-end reads (s.d. = 33,916,588).

**Table 1:** RNA-seq data used in the study.

Descriptive information on RNA-seq samples		
	Human	Primate
Data source	PRJNA530758; PRJNA551775; PRJNA551908; PRJNA551909	GSE96731; GSE144783
		GSE96732
n	56	53
Traits	53.57% AUD	16.98% Very high drinkers, 20.75% high drinkers
		13.2% Binge drinkers, 33.96% low drinkers and 15.09% Naïve
Brain regions	sPFC, NAc, BLA and CEA	PFC (Area 32), NAc core and CEA
Age	*M* = 57.34 (*s.d.* = 8.91)	4–11 years
Sex	76.79%	100% Male
Brain pH	6.60 (*s.d.* = 0.25)	NA
PMI	*M* = 32.81 (*s.d.* = 13.75)	< 1 h
Cigarette smoker	67.86%	0%

Primate samples lacked information on brain pH AUD includes both DSM-IV diagnoses of alcohol abuse (two thirds of AUD cases) and alcohol dependence (one third of AUD cases). PMI stands for post-mortem interval or the # of hours since death until brain tissue was frozen or processed.

Male primate samples came from four cohorts (4, 5, 7a and 7b) of Rhesus Macaques from the Monkey Alcohol Tissue Research Resource (www.MATRR.com). Primate brain samples contained analogous brain regions as the human data, including the: (1) PFC^15^ (cortical area 32; GSE96731), (2) NAc core^16^ (GSE144783) and (3) CEA^15^ (GSE96732). Note the PFC and CEA primate samples were from the same study^15^. Monkeys were housed individually and across cohorts had an age between 4–11 years and an average weight of 9.14 kg (s.d. = 1.24). The alcohol use paradigm was described previously^17^. Briefly, monkeys were trained to drink a 4% alcohol solution for 4 months. After this, monkeys were permitted to self-administer alcohol for over a year with 22 h of open access to alcohol. Primate alcohol consumption in this model is comparable to human alcohol in inviduals with AUD^18^. Primate samples had five drinking categories: controls (alcohol naïve), low drinkers, high drinkers, binge drinkers, or very high drinkers. To reduce multiple testing, we collapsed the top drinking categories into a single alcohol group and compared this group to the lowest drinking category (naïve controls in the NAc or the low drinking category in the PFC and CEA; note PFC and CEA samples had no naïve alcohol group). We removed samples with a normalized RNA-seq read count below two standard deviations of the group mean, which left a total of 81 primate brain samples (n_NAC_ = 23; n_CEA_ = 28; n_PFC_ = 30). All primate procedures were reviewed and approved by the Oregon National Primate Research Center IACUC and were in accordance with the Guide for the Care and Use of Laboratory Animals as well as the NIH guidelines for the care and use of laboratory animal animals. For more information on the primate RNA-seq data see Iancu et al.^15^ and Walter et al.^16^. Briefly, paired-end and stranded RNA libraries were prepared via the TruSeq RNA sample preparation kit. PFC and CEA data were ribo-depleted (RiboZero Gold rRNA depletion) and sequenced on the Illumina HiSeq 2000, whereas NAc data were PolyA selected and sequenced on the Illumina HiSeq 2500. In total, the primate brain samples had an average of 43,133,419 paired-end reads (s.d. = 14,096,098).

#### GWAS

Alternative mRNA splicing associations were inferred from GWAS summary statistics from a study on problematic alcohol use, which we refer to as AUD for simplicity. AUD in this GWAS was defined as a DSM-V AUD diagnosis, a DSM-IV alcohol dependence diagnosis, or a log_10_ transformed metric of the Alcohol Use Disorders Identification Tests—problem drinking items. This study used 435,563 individuals of European ancestry (Age range = 22–90)^11^ across three major cohorts: the (1) Million Veteran’s Project, (2) Psychiatric Genomics Consortium and (3) United Kingdom BioBank.

#### Data preparation

RNA-seq data were processed using a uniform pipeline. First, we investigated RNA-seq data quality using FastQC (https://www.bioinformatics.babraham.ac.uk/projects/fastqc/). We removed Illumina adapters and poor quality reads (reads < 36 bp long, leading or trailing reads < Phred score of 3 and allowing a maximum of 2 mismatches per read) using Trimmomatic (version 0.39)^19^. Then, we aligned trimmed reads to either the human hg19 genome or the Rhesus Macaque mmul_10 genome using STAR aligner version 2.5.3.a^20^. We followed the guidelines outlined by leafcutter (https://davidaknowles.github.io/leafcutter) to align RNA-seq reads and prepare data for differential splicing analyses. RNA-seq read alignment yielded an average of 78,955,738 paired-end reads in humans (s.d. = 29,804,777; *M*_Alignment_ = 86.16%; *M*_read_size_ = 188.36) and a mean of 34,551,920 paired–end reads in primates (s.d. = 8,202,258; *M*_Alignment_ = 79.71%; *M*_read_size_ = 127.59).

DNA genotypes from human RNA-seq data were ascertained via the SAMtools mpileup function as done previously^21^. Human genotypes derived from RNA-seq data were phased and imputed with Beagle version 5.1, which uses a probabilistic Hidden Markov Chain model that performs well for sequencing data with sparse genomic coverage^22^. We would like to caution the reader that Beagle was originally developed for genome-wide DNA variant data and not RNA-sequencing data. Our analyses used a few methods and criteria for quality control (QC) including: genotyping rate > 95%, minor allele frequency > 0.10, Hardy–Weinberg equilibrium > 1e-6, > 5 reads per sample, Phred Score > 20 and an imputation score > 0.3. The input for imputation was 40,878 called genotypes that were common among all samples and passed initial QC. These variants were imputed to 1000 Genomes Phase III all data, which resulted in 570,755 SNPs, 178,598 of which passed QC. These ~ 170 k variants were used for polygenic score and sQTL analyses. Note, that the 91.9% of these SNPs were present in the AUD GWAS, but that GWAS has 77.9 times more SNPs than the current study. Thus, we encourage the reader to use caution in interpreting our polygenic score and sQTL analyses given the limited number of individuals and the number of SNPs used.

### Analyses

#### Differential splicing

To detect alternative mRNA associations with AUD we used Leafcutter version 0.2.9^23^. Leafcutter is a powerful transcriptome-wide splicing method that uses a Dirichlet-multinomial generalized linear regression to identify differentially spliced genes. A differentially spliced gene generally is composed of multiple clusters, each of which includes various alternative splicing events, such as exon-skipping (see Fig. 1), intron retention, alternative acceptor or alternative donor splice sites, which we annotated with the Vertebrate Alternative Splicing and Transcription Database (https://vastdb.crg.eu/wiki/Main_Page). Each splicing event corresponds to a change in percent spliced in (ΔPSI or dPSI) metric. In our AUD analyses, a positive ΔPSI for an exon skipping event would suggest that an individual with AUD is more likely to skip a certain exon than someone without AUD. We utilized the default filtering parameters of Leafcutter that filtered out splicing clusters with < 5 samplers per intron, < 3 samples per group, and required at least 20 reads, which resulted in 18,685 unique genes across human brain regions. Human differential splicing analyses covaried for sex, age, brain pH, PMI, and smoking status. Note leafcutter performs analyses at the cluster level calculating a cluster *p*-value and then performs a Benjamini–Hochberg False Discovery (BH-FDR) multiple testing correction. Differentially spliced genes/clusters were those that survived a standard BH-FDR adjusted *p*-value < 0.05. We corrected p-values for multiple testing within brain regions and thus, our analyses do not account for multiple testing across tissues or samples. Since only 21 genes were differentially spliced in primates (BH-FDR < 0.05), we defined significant differential splicing with a nominal *p*-value threshold < 0.05. When possible, primate differential splicing analyses controlled for age (NAc sample). We assessed linear correlations of the ΔPSI across all significant alternative splicing events that were common across brain regions.

To assess the overlap between human and primate results we used a Fisher’s Exact test at the gene-level and restricted analyses to homologous genes identified by biomaRt^24^ and only used results from analogous regions of the brain (CEA, NAc, and PFC). In humans, we compared our differential splicing analyses with differentially expressed genes. Differential expression analyses leveraged featureCounts to count aligned RNA-seq reads and used DESeq2^25^ to determine differential expression. Differential expression analyses used the same covariates and p-value adjustment as differential splicing analyses. Previous differential splicing analyses of these data^7^ used rMATS^26^ that focuses on individual splicing events (rather than broader clusters within genes) and leverages a joint likelihood function combining binomial and normal distributions.

#### Polygenic scores

We investigated two questions with polygenic score analyses. First, did AUD cases have higher mean polygenetic scores for AUD than control samples? Second, are AUD polygenic scores associated with alternative mRNA splicing in the brain? Polygenic score hypotheses were tested using PRScice.2 (version 2.3.3)^27^. We elected to use standard polygenic score guidelines^28^. Specifically, we performed quality control on the base data, which was the AUD GWAS summary statistics (minor allele frequency > 0.01, remove duplicate and ambiguous SNPs). Our target data was the cleaned and imputed RNA-seq brain data (genotyping rate > 95%, minor allele frequency > 0.10, Hardy–Weinberg equilibrium < 1e-6, read depth > 5 reads per sample, Phred Score > 20 and imputation score > 0.3). We used the default parameters from PRScice.2, which removed variants in linkage disequilibrium (LD) with each other (clumping) and selected the most associated polygenic score via a p-value threshold approach using a certain number of genes that enhances prediction.

#### sQTLs

A splicing quantitative trait locus (sQTL) is a SNP that predicts alternative mRNA splicing associated with a trait. Similar to Li et al.^23^, we standardized excision-splicing ratios and then quantile normalized splicing data across individuals. Our analyses used default settings on MatrixQTL to find cis-acting sQTLs that may affect mRNA splicing in a nearby gene, which tests all SNPs within 1 megabase (Mb) of a genomic region. sQTLs were defined as a SNP associated with a differentially spliced gene that survived a BH-FDR correction for multiple testing per SNP. To determine whether sQTLs resided in specific regions of the genome we annotated sQTLs in 11 annotation categories from ANNOVAR (version 4.1)^29^. The annotation categories that were built on hg18 genome coordinates were updated to their corresponding hg19 values using CrossMap (version 0.5.1)^30^. Genetic analyses (polygenic score and sQTLs) controlled for sex, age, and two ancestral principal components.

#### Partitioned heritability

To test whether differentially spliced genes associated with AUD in the brain pointed to genetic mechanisms of alcohol misuse we performed a partitioned heritability analysis. We used LD score regression^31^ and created an annotated gene set of differentially spliced genes (BH-FDR < 0.05). To be consistent with our sQTL analyses, this included SNPs within 1 Mb of the start and stop site of a differentially spliced gene, which is similar to defaults on other splicing partitioned heritability mapping tools (e.g., Li et al.^32^). To determine the specificity of our findings, we tested the partitioned heritability of this gene-set with a negative control trait (Joint disorders found via: http://www.nealelab.is/uk-biobank) that used individuals of European ancestry and had similar sample size (n =  ~ 361,194) and trait heritability (*h*^2^_SNP_ = 0.0695) as AUD.

#### Splicing TWASs

We performed transcriptome-wide association studies (TWASs) via splicing SMulti-Xcan^33,34^, to assess how DNA associations predicted alternative mRNA splicing associations in human tissues. To increase power, we performed spicing TWASs on all of the 49 available Genotype-Tissue Expression (GTEx) database tissues (which included up to 838 human donors; https://www.gtexportal.org/home/) as done previously^2^. Since alternative mRNA splicing is tissue-specific, we also re-ran a splicing TWAS on AUD incorporating only the 13 GTEx brain tissues. The brain-specific splicing TWAS and the all-tissue splicing TWAS yielded fairly similar results (see Supplementary File S1). That is, 42.42% of the genes identified in the brain TWAS were identified in the all-tissue TWAS. Our manuscript focuses on the splicing TWAS using all 49 GTEx tissues, given that this analysis increased power and specifically boosted the number of significant genes over threefold compared to the splicing brain TWAS. SMultiXcan (the method used for our splicing TWAS) combines multiple regression and elastic neural networks to predict alternative mRNA splicing from cis-sQTLs. This method accounts for linkage disequilibrium (LD) of European ancestry using the 1000 Genomes Phase 3 data. Our study assessed the convergence between the splicing TWAS on AUD and the differentially spliced genes in the brain associated with AUD. Of the overlapping genes, we assessed SNP associations mapped to these genes that were associated with other traits via https://www.ebi.ac.uk/gwas/. For these genes that also had a significant sQTL we evaluated the LD between the lead sQTL SNP (smallest p-value for the gene) with the SNP listed in the GWAS catalog using LDlink (European Ancestry; https://ldlink.nci.nih.gov/?tab=home). Lastly, we investigated how splicing associations generalized across substance use traits by correlating splicing TWAS results from three other GWASs: cigarettes per day (n = 263,954)^35^, opioid use disorder (n = 82,707)^36^ and cannabis use disorder (n = 374,287)^37^.

## Results

### Polygenic scores associated with AUD and alternative mRNA splicing

**Figure 2:**
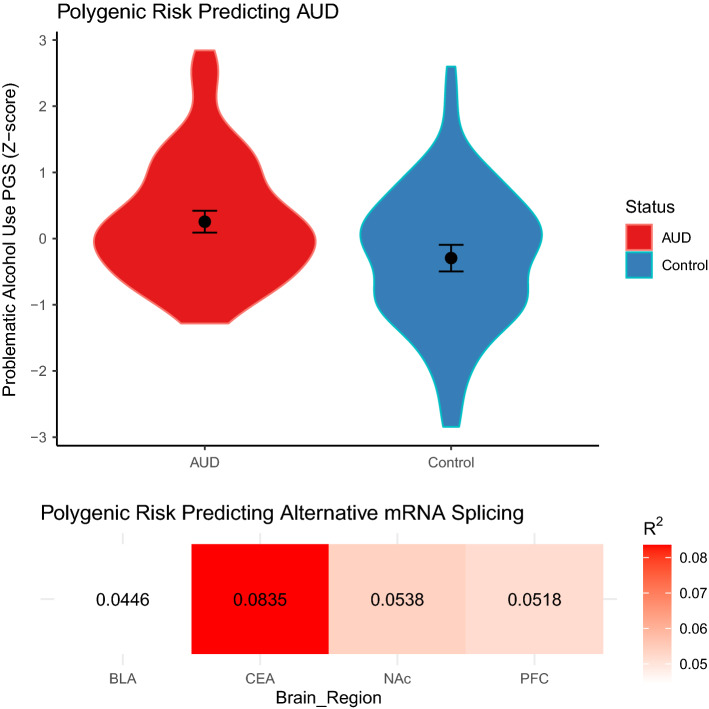
Genetic risk for AUD and alternative mRNA Splicing. (**A**) Violin plot showing polygenic score distributions of AUD between individuals with AUD and controls. Mean and standard error are shown. *P*-value thresholds were 0.0183, 0.1016, 0.3522 and 0.0187 for the BLA, CEA, NAc and PFC, respectively. Scores were combined across brain regions and tested between AUD cases and controls. (**B**) Heat matrix showing the amount of variance explained (R^2^) by polygenic score analyses of differential splicing results for each brain region. Principal components (PC) analysis was used to distil differential splicing results into a single metric (1st PC). Polygenic score p-value thresholds were 5.e−5, 0.006, 5e−5 and 0.0012 for the BLA, CEA, NAc and PFC, respectively.

Polygenic score analyses indicated that individuals with AUD had an increased average polygenic risk for AUD than healthy controls (*p* = 0.00003; Fig. 2). Polygenic scores were linked to alternative mRNA splicing in the CEA (*p* = 0.035), trending in the NAc and PFC (all *p* < 0.099) and non-significant in the BLA (*p* = 0.119; Fig. 2; see Supplementary Files S2–S3). Given limitations of sample size and sparse genomic coverage, we encourage the reader to take caution in interpreting results from our polygenic score analyses.

### Differential splices genes between AUD vs controls

**Figure 3:**
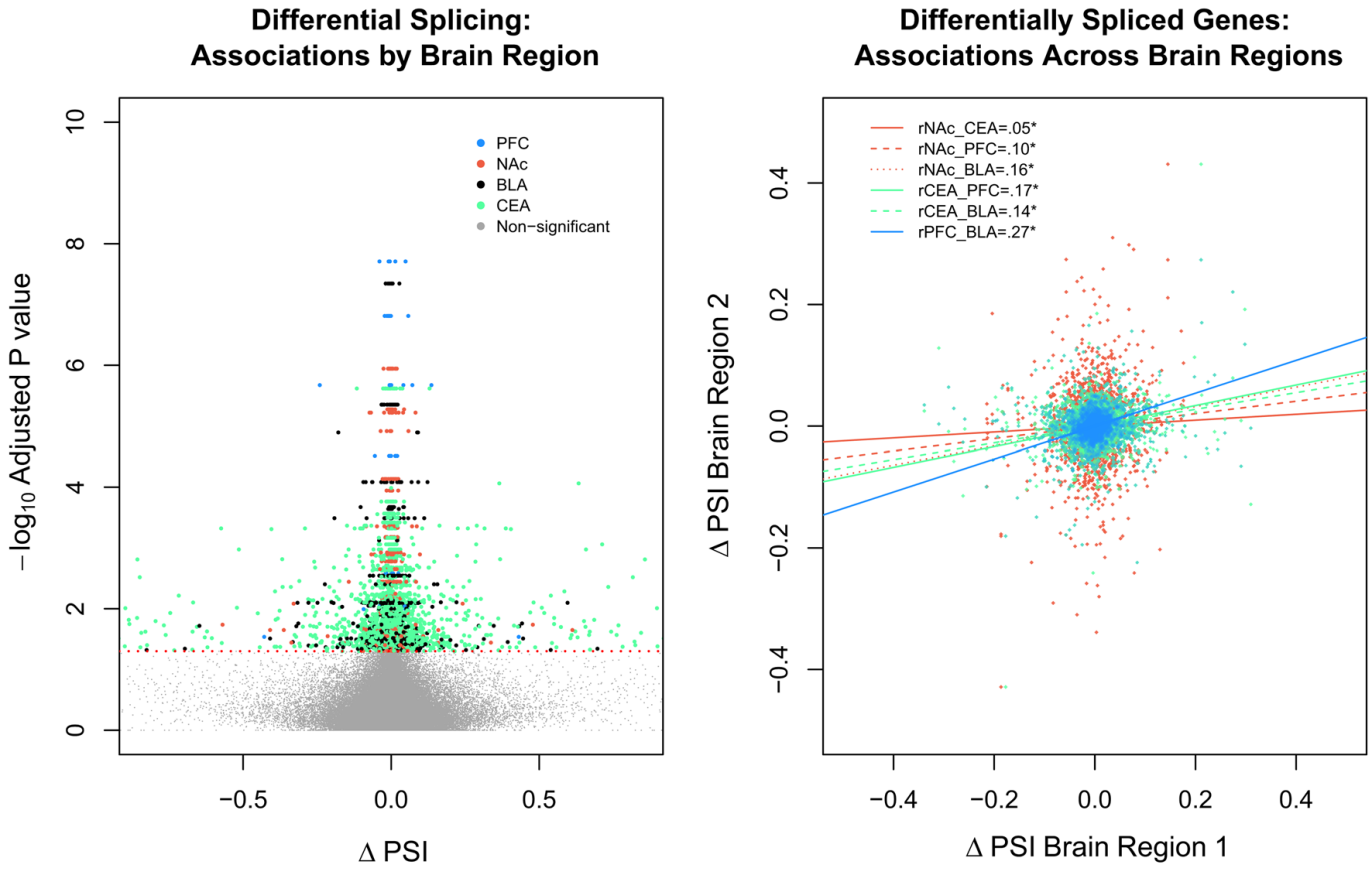
Alternative mRNA splicing associations with AUD by brain region. (**A**) Volcano plot displaying differentially spliced genes between individuals with AUD and controls for each brain region. (**B**) Scatter plot showing differential splicing associations across brain regions from differentially spliced genes. Note ΔPSI stands for the change in percent-spliced-in and that each colored dot represents a specific splicing event in a cluster from a significantly differentially spliced gene (*p*_adj_ < 0.05).

In total, we found 713 differentially spliced genes in 740 clusters encompassing 5118 unique splicing events associated with AUD (see Fig. 3 and Supplementary File S4). Note, we identified more clusters than genes in these analyses as some genes had multiple clusters that were differentially spliced and because other clusters corresponded to a gene region without an official annotated gene symbol. Similar to previous analyses with these data, 92.3% of the reported differentially spliced genes associated with AUD^7^, were at least nominally significant in our analyses. We also identified exon skipping as the most frequent splicing event (53.9%) and found alternative splice donor events (4.0%) to be the least frequent. Differentially spliced genes were not enriched for gene ontological processes (all *p*_adj_ > 0.39), but several addiction genes were found to be differentially spliced, including *ALDH3A2, CAMK2D, CAMKK2, GRIA2*, *GRK4, GRK6, HDAC3, PPP2R1B,* and *PRKACB* (see Supplementary Figs. S3–S4). The *GRIA2* gene showed differential splicing in a putative ‘flip flop’ splicing site (see Supplementary Fig. S5), which alters the rate of AMPA receptor opening^38,39^ and has been implicated with chronic alcohol use in primates^40^. We found 53 differentially expressed genes associated with AUD (all *p*_adj_ < 0.05; see Supplementary File S5). Note, no differentially expressed gene was differentially spliced.

### Conservation of splicing associations across humans and primates

Investigating analogous brain regions in Macaques, we found that AUD differentially *spliced* genes tended to also demonstrate differential splicing in primate models of chronic alcohol use (see Supplementary Fig. S6). This overlap was more than we expected by chance, OR = 1.38, 95% CI [1.06, 1.77], *p* = 0.0126. We found significant, yet small, correlations of splicing events across brain regions in humans (r = 0.05–0.27; see Fig. 3), yet only 23 out of 713 genes (~ 3%) were differentially spliced across brain regions (see Supplementary Fig. S7). In the primate data, we found significant positive associations of differential splicing across brain regions when using the same individual primate samples (e.g., same monkeys but different tissues: PFC and CEA: *r* = 0.10, *p* = 2e−16). However, splicing associations across brain regions were negative when looking across different primate samples (NAc with the PFC: r =  − 0.04 and NAc with CEA r =  − 0.08, all *p* < 0.002). Altogether, these results suggest alcohol-related mRNA splicing is largely tissue-specific and that overlap across regions may be due to the same samples/individuals.

### Genetic variation correlates with AUD-related alternative splicing

**Figure 4:**
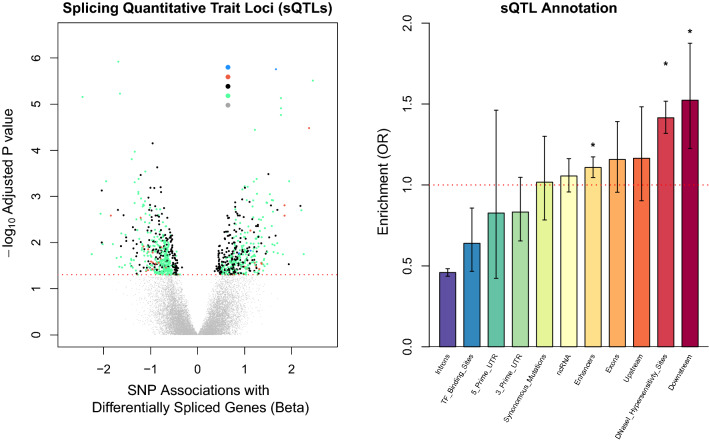
Individual DNA markers linked with alternative mRNA splicing events associated with AUD. (**A**) Volcano plot showing results from our sQTL analyses. Each dot above the dashed red line represents a significant (*p*_adj_ < 0.05) SNP association with a differentially spliced gene. (**B**) Bar plot showing the genomic regions enriched for significant sQTL associations. * indicated that a certain genomic region survived correction for multiple testing (*p*_adj_ < 0.05).

Next, we tested for sQTLs, or whether specific genetic variants were associated with the differentially spliced genes associated with AUD. In total, we found 6,463 unique sQTLs linked with 170 different genes (*p*_adj_ < 0.05; see Fig. 4 and Supplementary File S6). Drug metabolism (*CYP2C19* and *CYP2C9*) intracellular signaling (*GRK4, GRK6, HDAC3, PRKACB,* and *MAPK3K6*) and calcium ion channel genes (*CACNA1A, CACNA1G, CACNB2,* and *KCNMA1*) had sQTL(s). Exon skipping events in the *CACNA1A* and *KCNMA1* genes corresponded to certain gene formations that differentially alter vesicular release^41^ and activation of Ca^+^ channels^42^. Most sQTLs were located in intergenic regions (52.3%) or introns (36.1%), but we only identified sQTL enrichment among DNaseI hypersensitivity sites, enhancer regions, and downstream locations of protein-coding genes (see Fig. 4).

### The heritability of AUD is enriched for differentially spliced genes

**Figure 5:**
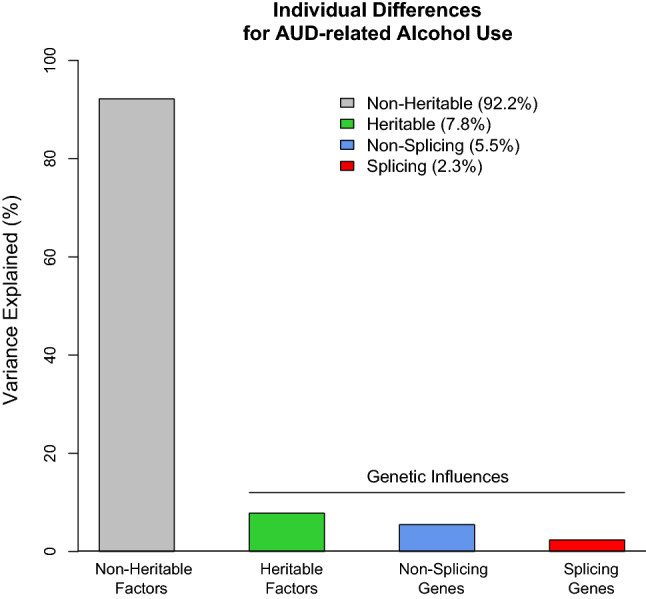
SNPs within and around differentially spliced genes contribute to the heritability of AUD. Heritable factors include the observed heritability from LD score regression analyses. Splicing genes include all biallelic SNPs within and 1 Mb around the transcription start and end site of differentially spliced genes associated with AUD in the brain.

We further investigated the role of alternative splicing for the genetic basis of AUD. Using LDscore regression we observed that heritable influences explained 7.81% of the individual differences in AUD. Our partitioned heritability analyses revealed that SNPs in and around differentially spliced genes accounted for 30% of the genetic risk for AUD (OR = 1.349, se = 0.064, *p* = 6.46e−7; see Fig. 5), but not for our negative control trait (Joint disorders, *p* = 0.161).

### Splicing TWASs are associated with substance use traits

We found 311 splicing TWAS associations with disordered alcohol use (*p*_adj_ < 0.05; 215 unique genes; see Supplementary File S7), which were enriched for alcohol dehydrogenase activity (*p*_adj_ = 3.23e−10). Seven of the TWAS splicing genes were also differentially spliced genes in post-mortem brain tissue (*GRK4, KLHDC8B, PDS5A, PSMD7, TMEM184B, VRK2,* and *WDR27*). The role of these genes in the pathophysiology of AUD is largely unknown. Previous research suggests that SNPs mapped to these genes are associated with substance use traits, neuropsychiatric illnesses, and neurological endophenotypes as well as other unrelated traits (see Supplementary File S8). Of note, our lead sQTLs for the *GRK4* (rs2858038) and *KLHDC8B* (rs3819325) genes were in LD with SNPs associated with human cigarettes per day (rs2960306, R^2^ = 0.29) and smoking cessation (rs7617480, R^2^ = 0.07)^35^. To investigate potential shared genetic processes across substance use, we correlated significant splicing TWAS associations across three substance use traits: cigarettes per day^35^, opioid use disorder^36^, and cannabis use disorder^37^. Using the 1397 significant splicing TWAS associations across substance use traits (BH-FDR < 0.05; 923 unique genes; see Supplementary File S9), we found substantial overlap—especially among disordered substance use (all r > 0.38; see Supplementary Fig. S8).

## Discussion

Our exploratory study hypothesized that (1) polygenic scores of AUD would be higher in those with AUD than controls, (2) individual SNPs would be associated with abnormal alternative mRNA splicing in the brain, and (3) DNA variants around differentially spliced genes would contribute to the heritability of AUD.

Our exploratory study found novel splicing associations with AUD. We found support for our three hypotheses, such that: (1) polygenic scores were increased in those with AUD, (2) specific SNPs were associated with abnormal mRNA splicing events in multiple brain regions and (3) DNA variants in and around differentially spliced genes contributed to the heritability of AUD. Altogether, we used a handful of methods that provided evidence implicating genetic factors in AUD-related alternative mRNA splicing. These data add another layer to the neuroepigenetic understanding of compulsive alcohol use.

The takeaways from our study are consistent with previous analyses of these data. Both our study and the Van Booven et al., study^7^ found: (1) thousands of differentially spliced events between individuals with AUD and controls, with (2) largely tissue-specific findings and (3) different magnitudes of splicing associations by brain region as well as (4) more significant splicing associations with AUD than differential expression associations. Also, at the genetic level, we identified an order of magnitude more sQTLs than the previously reported (and validated) expression QTLs (eQTLs) with AUD^14^. These results are consistent with previous analyses suggesting alternative mRNA splicing elicits robust genetic and neurotranscriptional correlates with psychiatric traits^2^ and calls for additional research to better characterize the gene isoform architecture of mental illness and substance abuse.

Extending research on other neurological traits^32,43^, we show that individual genetic markers (sQTLs) and polygenic risk underlie alternative mRNA splicing associated with AUD. Similar to other research^44^, we found that sQTLs were enriched among DNaseI hypersensitivity sites, corroborating that loose chromatin regions are hotspots for alternative mRNA splicing regulation. Previous splicing studies used a single tissue type^2,43–45^. Our study suggests that future work should consider multiple tissues when possible—as the genetic links with splicing events may differ by brain region.

Splicing associations with AUD occurred in genes involved with neurotransmission, intracellular signaling, and drug/alcohol metabolism. Most alternative mRNA splicing events were uncharacterized, but a few of the ion channel (*CACNA1A, KCNMA1*) and glutamate receptor (*GRIA2*) associations seemed to affect synaptic neurotransmission. For instance, in the BLA, we found that individuals with AUD were more likely to have an exon skipping event of the *GRIA2* flip exon (exon 14), which is associated with longer glutamate receptor opening and consistent with the BLA pathology in alcohol use^46–48^.

We found *preliminary* evidence that alternative mRNA splicing could play a more general role in a common genetic liability of substance use disorders and psychopathology. Our study revealed moderate splicing associations across disordered and problematic drug use as well as tobacco consumption, via splicing TWASs. Furthermore, the sQTLs underlying AUD-related differential splicing in the brain were correlated with DNA variants previously implicated in tobacco consumption, mental illness, and cognitive functioning. Additionally, differentially spliced genes correlated with AUD in our analyses were also linked with brain splicing associations with autism spectrum disorder and schizophrenia^2^, which included glutamate receptor (*GRIA2*) and calcium signaling genes (*CACNA1G, CAMK2D,* and *CAMKMT*) as well as intracellular processes (*AKAP13, ARPP21, PRKACB,* and *PTPRS*) and synaptic plasticity genes (*ARHGEF10L*, *ARHGEF4, CLASP2, GAPVD1, NTNG2, SUN1,* and *TPM3*).

While our study sought to characterize the genetic roots of alcohol-related alternative mRNA splicing, we cannot dismiss the potential for alcohol-induced differential splicing. We found that many of the differentially spliced genes associated with AUD were also differentially spliced in primate models of chronic binge drinking. Notably, only five of these overlapping genes from analogous brain regions had a sQTL (2.9% of sQTLs). This may suggest that both genetic and alcohol-related mechanisms underlie alternative mRNA splicing in the brain.

The current study should be interpreted in the context of the following limitations. First and foremost, our study used small RNA-seq samples that lacked direct replication or validation samples. Results from small samples may incur unreliable effect size estimates and could be more prone to a winner’s curse. But, we sought to assess the reproducibility of our findings by utilizing multiple tissue and data types as well as cross-referencing findings with GWASs that contain much larger samples. Polygenic scores and differential splicing associations with AUD are likely both associated with chronic alcohol use and thus may confound interpretations of pure genetic links with mRNA splicing in human brain tissue. Polygenic scores used PRScise2, which chooses a threshold that maximizes prediction and over-fits the data. Partitioned heritability analyses indicated that alternative mRNA splicing explained a significant amount of the heritability, but this is still ~ 2% of the total individual differences in AUD and may include non-splicing related DNA variants. The GWASs used in our study included some overlapping participants (e.g., UK BioBank and Million Veterans Project) and were limited to individuals of European Ancestry. Human brain data had long post-mortem intervals, which could lead to poor RNA quality and high RNA degradation—potentially biasing results towards shorter transcripts. Primate brain samples were not perfectly matched to the human data, as primates were limited to males, neuroanatomical location diverged (especially in PFC), and often the specific splicing events within genes differed across species. Lastly, the results from our study were based on computational analyses and require experimental follow-up to buttress the confidence of these findings and their role in AUD. Notwithstanding these limitations, our study added context to our genetic and neurobiological understanding of AUD.

## Supplementary Information

Supplementary Information 1.

Supplementary Information 2.

Supplementary Information 3.

Supplementary Information 4.

Supplementary Information 5.

Supplementary Information 6.

Supplementary Information 7.

Supplementary Information 8.

Supplementary Information 9.

Supplementary Information 10.

## Data Availability

The datasets analyzed during the current study are available in Sequence Read Archive (Human brain data: PRJNA530758, PRJNA551775, PRJNA551909 and PRJNA551908) or the Gene Expression Omnibus (GSE96731, GSE144783 and GSE96732). All procedures used in this study are in accordance with ARRIVE guidelines.

## References

[1] Pan Q, Shai O, Lee LJ, Frey BJ, Blencowe BJ (2008). Deep surveying of alternative splicing complexity in the human transcriptome by high-throughput sequencing. Nat. Genet..

[2] Gandal MJ, Zhang P, Hadjimichael E, Walker RL, Chen C, Liu S (2018). Transcriptome-wide isoform-level dysregulation in ASD, schizophrenia, and bipolar disorder. Science.

[3] Donadoni M, Cicalese S, Sarkar DK, Chang SL, Sariyer IK (2019). Alcohol exposure alters pre-mRNA splicing of antiapoptotic Mcl-1L isoform and induces apoptosis in neural progenitors and immature neurons. Cell Death Dis..

[4] Signor S, Nuzhdin S (2018). Dynamic changes in gene expression and alternative splicing mediate the response to acute alcohol exposure in *Drosophila melanogaster*. Heredity (Edinb.).

[5] Farris SP, Arasappan D, Hunicke-Smith S, Harris RA, Mayfield RD (2015). Transcriptome organization for chronic alcohol abuse in human brain. Mol. Psychiatry.

[6] Lee C, Mayfield RD, Harris RA (2014). Altered gamma-aminobutyric acid type B receptor subunit 1 splicing in alcoholics. Biol. Psychiatry.

[7] Van Booven D, Mengying L, Sunil Rao J, Blokhin IO, Dayne Mayfield R, Barbier E (2021). Alcohol use disorder causes global changes in splicing in the human brain. Transl. Psychiatry.

[8] Kapoor M, Wang JC, Farris SP, Liu Y, McClintick J, Gupta I, Meyers JL, Bertelsen S, Chao M, Nurnberger J, Tischfield J (2019). Analysis of whole genome-transcriptomic organization in brain to identify genes associated with alcoholism. Transl. Psychiatry.

[9] Huggett SB, Stallings MC (2020). Genetic architecture and molecular neuropathology of human cocaine addiction. J. Neurosci..

[10] Gandal MJ, Haney JR, Parikshak NN, Leppa V, Ramaswami G, Hartl C, Schork AJ, Appadurai V, Buil A, Werge TM, Liu C (2018). Shared molecular neuropathology across major psychiatric disorders parallels polygenic overlap. Science.

[11] Zhou H, Sealock JM, Sanchez-Roige S, Clarke TK, Levey DF, Cheng Z (2020). Genome-wide meta-analysis of problematic alcohol use in 435,563 individuals yields insights into biology and relationships with other traits. Nat. Neurosci..

[12] Kranzler HR, Zhou H, Kember RL, Vickers Smith R, Justice AC, Damrauer S (2019). Genome-wide association study of alcohol consumption and use disorder in 274,424 individuals from multiple populations. Nat. Commun..

[13] Palmer RH, McGeary JE, Heath AC, Keller MC, Brick LA, Knopik VS (2015). Shared additive genetic influences on DSM-IV criteria for alcohol dependence in subjects of European ancestry. Addiction.

[14] Rao X, Thapa KS, Chen AB, Lin H, Gao H, Reiter JL (2021). Allele-specific expression and high-throughput reporter assay reveal functional genetic variants associated with alcohol use disorders. Mol. Psychiatry.

[15] Iancu OD, Colville A, Walter NA, Darakjian P, Oberbeck DL, Daunais JB, Zheng CL, Searles RP, McWeeney SK, Grant KA, Hitzemann R (2018). On the relationships in rhesus macaques between chronic ethanol consumption and the brain transcriptome. Addict. Biol..

[16] Walter N, Cervera-Juanes R, Zheng C, Darakjian P, Lockwood D, Cuzon-Carlson V, Ray K, Fei S, Conrad D, Searles R, Grant K (2021). Effect of chronic ethanol consumption in rhesus macaques on the nucleus accumbens core transcriptome. Addict. Biol..

[17] Grant KA, Leng X, Green HL, Szeliga KT, Rogers LS, Gonzales SW (2008). Drinking typography established by scheduled induction predicts chronic heavy drinking in a monkey model of ethanol self-administration. Alcohol. Clin. Exp. Res..

[18] Baker EJ, Farro J, Gonzales S, Helms C, Grant KA (2014). Chronic alcohol self-administration in monkeys shows long-term quantity/frequency categorical stability. Alcohol. Clin. Exp. Res..

[19] Bolger AM, Lohse M, Usadel B (2014). Trimmomatic: A flexible trimmer for Illumina sequence data. Bioinformatics.

[20] Dobin A, Davis CA, Schlesinger F, Drenkow J, Zaleski C, Jha S (2013). STAR: Ultrafast universal RNA-seq aligner. Bioinformatics.

[21] Saad MH, Rumschlag M, Guerra MH, Savonen CL, Jaster AM, Olson PD (2019). Differentially expressed gene netowrks, biomarkers long noncoding RNAs and shared responses with cocaine identified in the midbrains of human opioid abusers. Sci. Rep..

[22] Browning BL, Zhou Y, Browning SR (2018). A one-penny imputed genome from next-generation reference panels. Am. J. Hum. Genet..

[23] Li YI, Knowles DA, Humphrey J, Barbeira AN, Dickinson SP, Im HK (2018). Annotation-free quantification of RNA splicing using LeafCutter. Nat. Genet..

[24] Smedley D, Haider S, Ballester B, Holland R, London D, Thorisson G (2009). BioMart–biological queries made easy. BMC Genomics.

[25] Love MI, Huber W, Anders S (2014). Moderated estimation of fold change and dispersion for RNA-seq data with DESeq2. Genome Biol..

[26] Shen S, Park JW, Lu ZX, Lin L, Henry MD, Wu YN, Zhou Q, Xing Y (2014). rMATS: Robust and flexible detection of differential alternative splicing from replicate RNA-Seq data. Proc. Natl. Acad. Sci..

[27] Choi SW, O'Reilly PF (2019). PRSice-2: Polygenic Risk Score software for biobank-scale data. Gigascience.

[28] Choi SW, Mak TS-H, Oreilly PF (2020). Tutorial: A guide to performing polygenic risk score analyses. Nat. Protoc..

[29] Wang K, Li M, Hakonarson H (2010). ANNOVAR: Functional annotation of genetic variants from high-throughput sequencing data. Nucleic Acids Res..

[30] Zhao H, Sun Z, Wang J, Huang H, Kocher JP, Wang L (2014). CrossMap: A versatile tool for coordinate conversion between genome assemblies. Bioinformatics.

[31] Bulik-Sullivan BK, Loh PR, Finucane HK, Ripke S, Yang J, Patterson N, Daly MJ, Price AL, Neale BM (2015). LD Score regression distinguishes confounding from polygenicity in genome-wide association studies. Nat. Genet..

[32] Li YI, Wong G, Humphrey J, Raj T (2019). Prioritizing Parkinson's disease genes using population-scale transcriptomic data. Nat. Commun..

[33] Barbeira AN, Pividori M, Zheng J, Wheeler HE, Nicolae DL, Im HK (2019). Integrating predicted transcriptome from multiple tissues improves association detection. PLOS Genet..

[34] Barbeira AN, Dickinson SP, Bonazzola R, Zheng J, Wheeler HE, Torres JM (2018). Exploring the phenotypic consequences of tissue specific gene expression variation inferred from GWAS summary statistics. Nat. Commun..

[35] Liu M, Jiang Y, Wedow R, Li Y, Brazel DM, Chen F (2019). Association studies of up to 1.2 million individuals yield new insights into the genetic etiology of tobacco and alcohol use. Nat. Genet..

[36] Zhou H, Rentsch CT, Cheng Z, Kember RL, Nunez YZ, Sherva RM (2020). Association of OPRM1 functional coding variant with opioid use disorder: A genome-wide association study. JAMA Psychiat..

[37] Johnson EC, Demontis D, Thorgeirsson TE, Walters RK, Polimanti R, Hatoum AS (2020). A large-scale genome-wide association study meta-analysis of cannabis use disorder. Lancet Psychiatry.

[38] Schlesinger F, Tammena D, Krampfl K, Bufler J (2005). Desensitization and resensitization are independently regulated in human recombinant GluR subunit coassemblies. Synapse.

[39] Penn AC, Balik A, Wozny C, Cais O, Greger IH (2012). Activity-mediated AMPA receptor remodeling, driven by alternative splicing in the ligand-binding domain. Neuron.

[40] Acosta G, Freidman DP, Grant KA, Hemby SE (2011). Alternative splicing of AMPA subunits in prefrontal cortical fields of cynomolgus monkeys following chronic ethanol self-administration. Front. Psychiatry.

[41] Chen L, Tian L, MacDonald SH, McClafferty H, Hammond MS, Huibant JM (2005). Functionally diverse complement of large conductance calcium- and voltage-activated potassium channel (BK) alpha-subunits generated from a single site of splicing. J. Biol. Chem..

[42] Heck J, Parutto P, Ciuraszkiewicz A, Bikbaev A, Freund R, Mitlöhner J (2019). Transient confinement of Ca(V)2.1 Ca(2+)-channel splice variants shapes synaptic short-term plasticity. Neuron.

[43] Raj T, Li YI, Wong G, Humphrey J, Wang M, Ramdhani S (2018). Integrative transcriptome analyses of the aging brain implicate altered splicing in Alzheimer's disease susceptibility. Nat. Genet..

[44] Takata A, Matsumoto N, Kato T (2017). Genome-wide identification of splicing QTLs in the human brain and their enrichment among schizophrenia-associated loci. Nat. Commun..

[45] Zhang X, Joehanes R, Chen BH, Huan T, Ying S, Munson PJ (2015). Identification of common genetic variants controlling transcript isoform variation in human whole blood. Nat. Genet..

[46] Pei W, Huang Z, Wang C, Han Y, Park JS, Niu L (2009). Flip and flop: A molecular determinant for AMPA receptor channel opening. Biochemistry.

[47] Gilpin NW, Herman MA, Roberto M (2015). The central amygdala as an integrative hub for anxiety and alcohol use disorders. Biol. Psychiatry.

[48] Janak PH, Tye KM (2015). From circuits to behaviour in the amygdala. Nature.

