# Supplementary material for: Genome- and transcriptome-wide splicing associations with alcohol use disorder
Source: Sci Rep. 2023 Mar 9;13:3950. doi: 10.1038/s41598-023-30926-z (PMC9998611; doi:10.1038/s41598-023-30926-z)
Supplement: Supplementary file 1 — Supplementary Information 1. [file 41598_2023_30926_MOESM1_ESM.docx]

Supplementary Information

**Supplementary Figure S1** Principal Components Plot Showing Genetic Ancestry of Post-mortem RNA-sequencing data


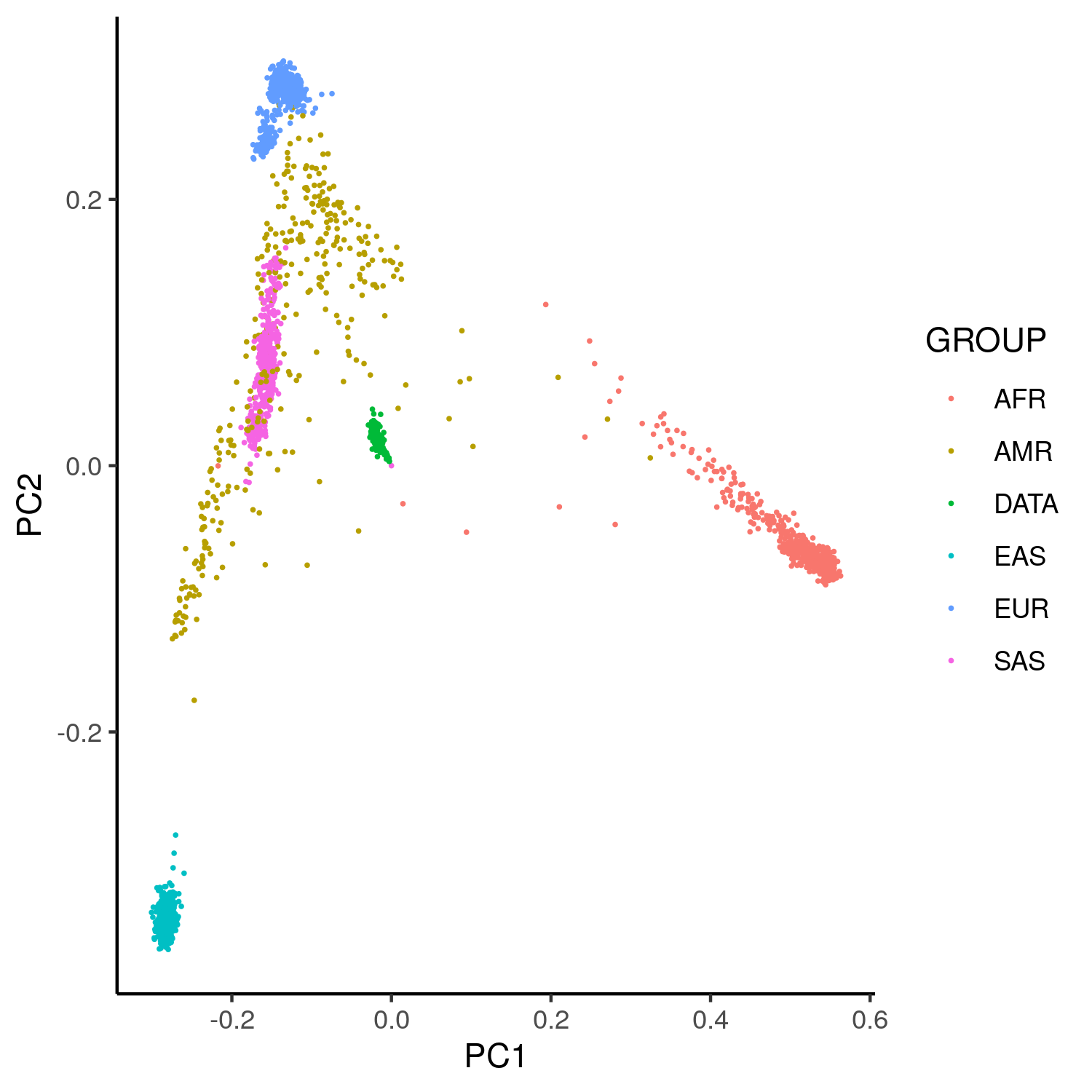


Each dot represents an individual sample and is color-coded by ancestry. Note that our RNA-seq data are shown in green, whereas all other colors represent ancestries from reference samples in 1K Genomes. AFR = African; AMR = Ad mixed American; EAS = Eastern Asian; EUR = European; SAS = South Asian.

**Supplementary Figure S2** Distributions and Associations of AUD Covariates

**
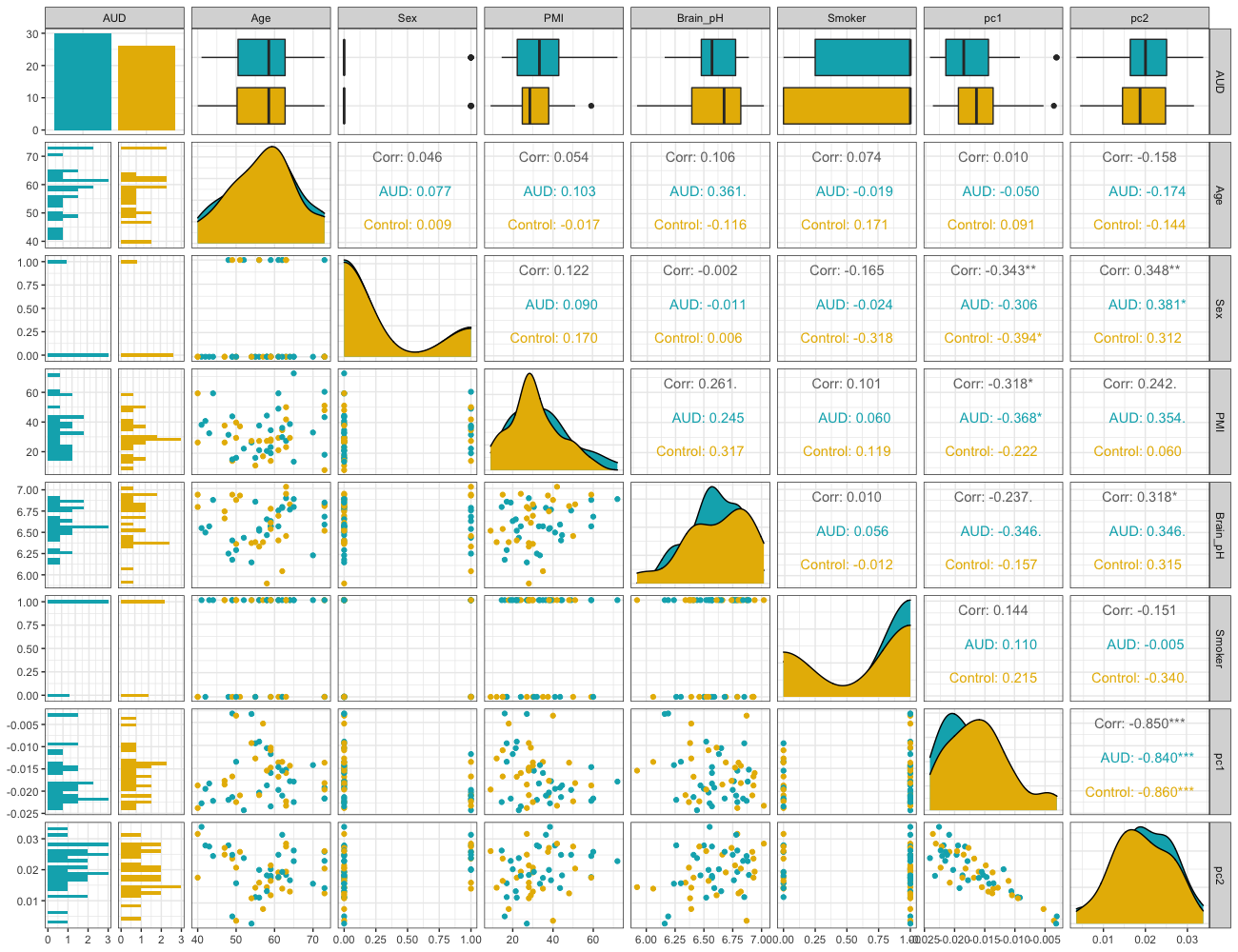
**

Upper diagonal shows box plots and correlation output of covariate associations by condition. The diagonal shows the density distributions of each covariate by condition. The lower diagonal includes scatter plots and shows a more nuanced representation of the value spread for each variable by condition. * p < 0.05, ** p < 0.01; *** p < 0.001

**Supplementary Figure S3** Differentially Spliced Addiction Genes in the CEA


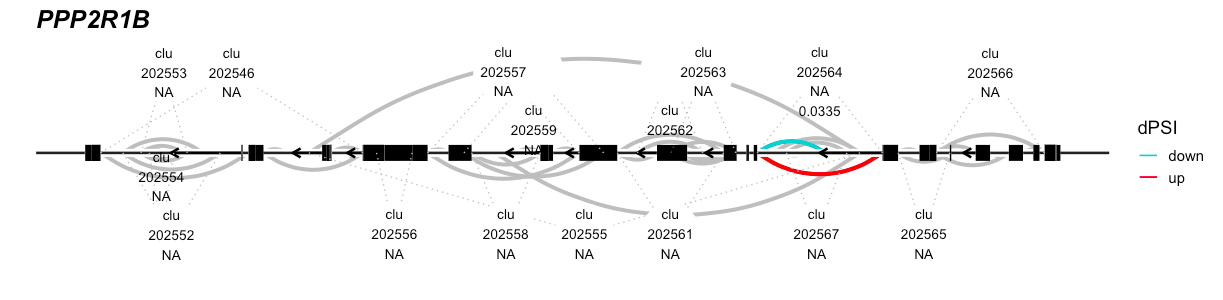

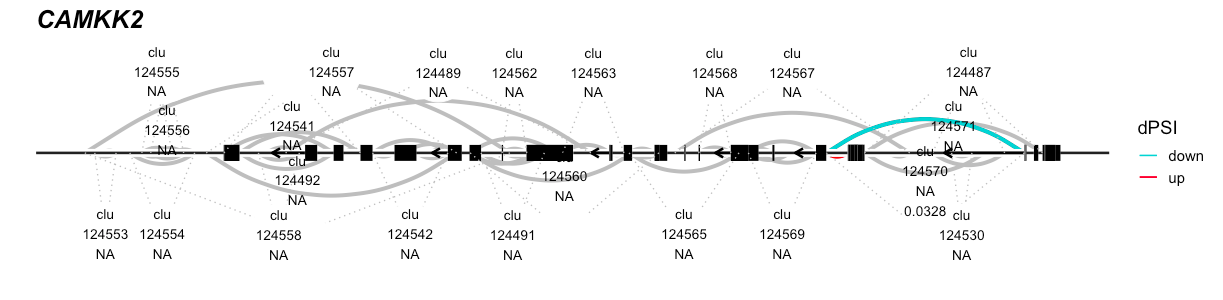

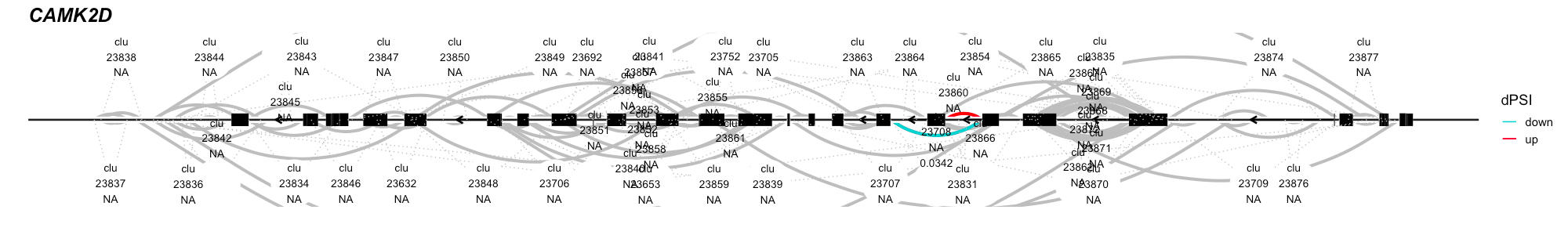

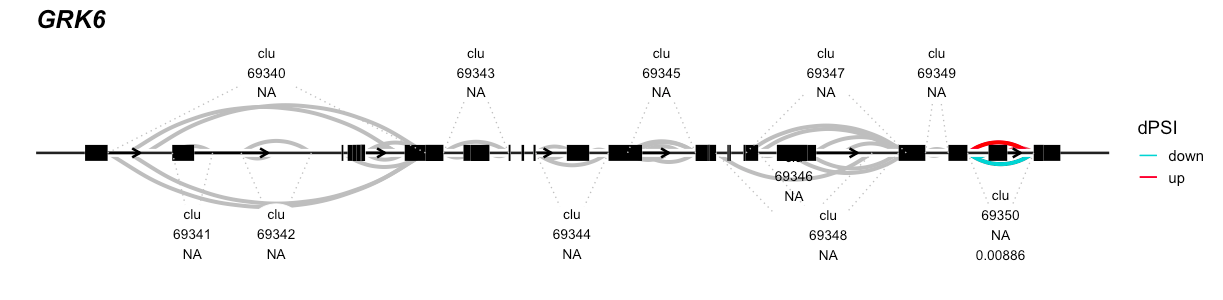


Differentially spliced clusters in the genes are labeled and emphasized in color. Red arcs show increased splicing in individuals with AUD (e.g., gene exons are more likely to be connected in AUD than controls). Cyan arcs represent decreased splicing in individuals with AUD (e.g., gene exons are more likely to be connected in controls than in individuals with AUD).

**Supplementary Figure S4** Differentially Spliced Addiction Genes in the BLA


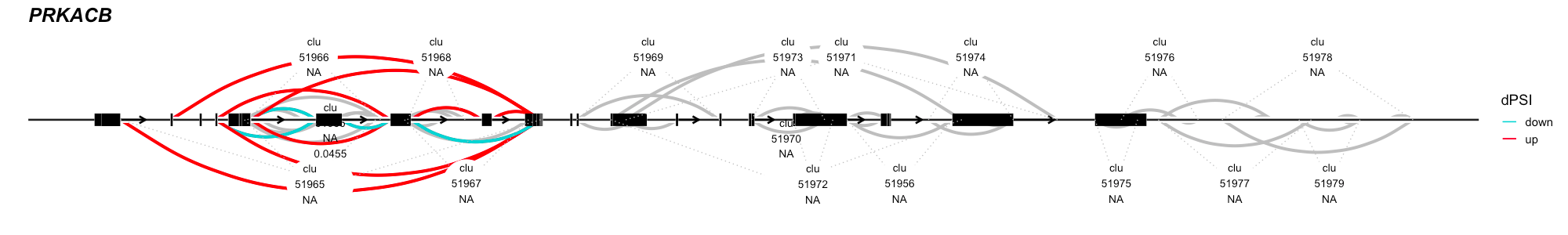

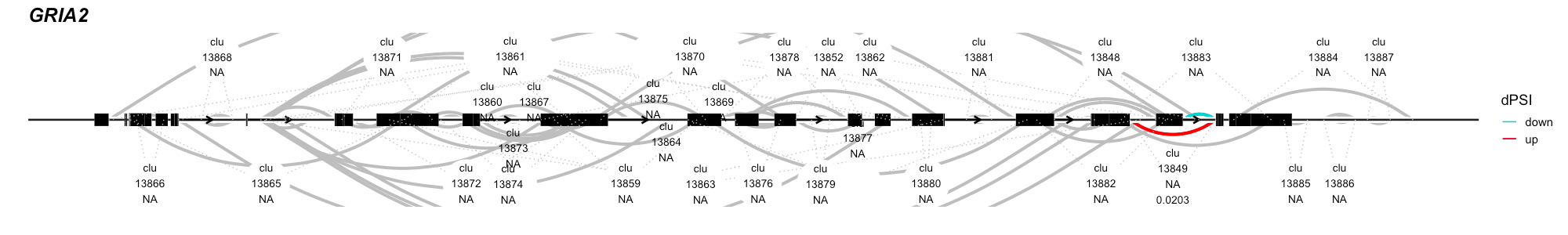

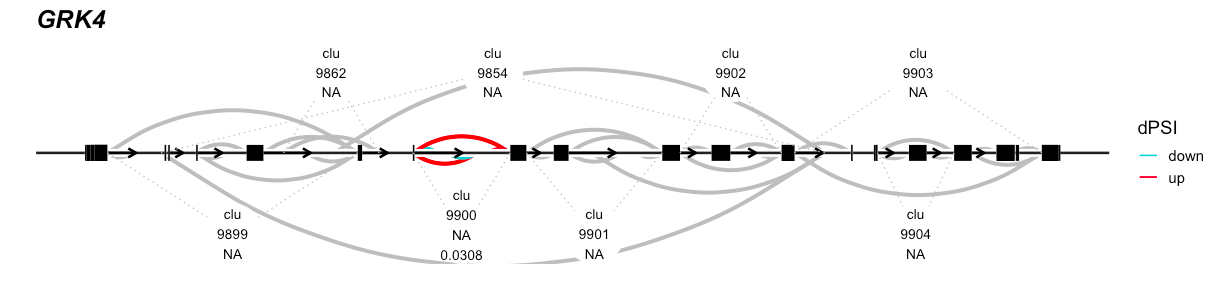

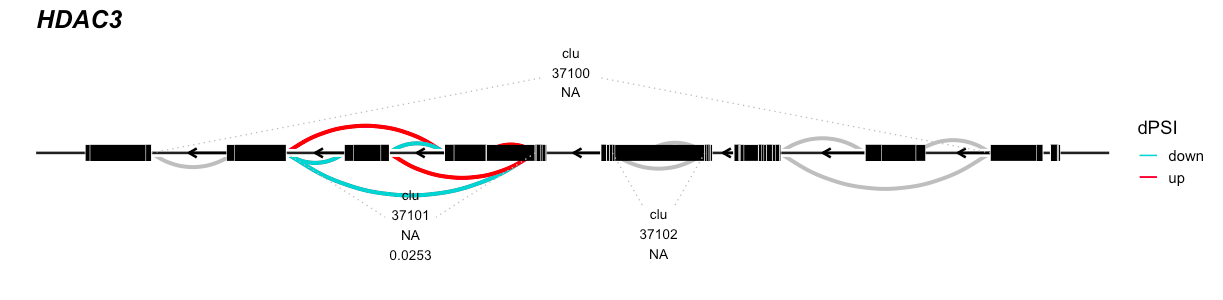


Differentially spliced clusters in the genes are labeled and emphasized in color. Red arcs show increased splicing in individuals with AUD (e.g., gene exons are more likely to be connected in AUD than controls). Cyan arcs represent decreased splicing in individuals with AUD (e.g., gene exons are more likely to be connected in controls than in individuals with AUD).

**Supplementary Figure S5** Differentially Splicing of *GRIA2* in BLA Associated with AUD


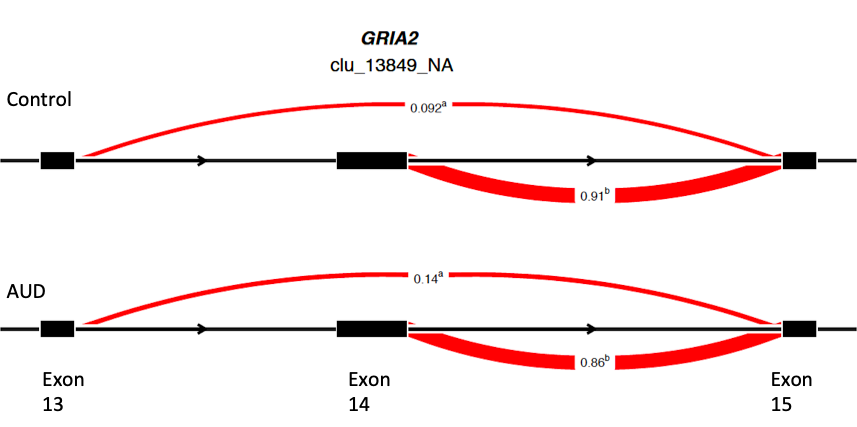


Exons are represented by black boxes and introns by black lines. Red arcs delineate splicing events and the numbers within each arc represent how often the exons are spliced together in either a control (top) or an AUD brain (bottom). Note that individuals with AUD are more likely to skip exon 14 in the BLA, which corresponds to an annotated splice site that influences AMPA receptor opening.

**Supplementary Figure S6** Differentially Spliced Genes Across Human AUD and Chronic Alcohol Use in Monkeys

Heatmap showing differentially spliced genes associated with human AUD and monkey alcohol consumption. Note differential splicing in monkeys used a nominal p-value cutoff of p < 0.05 due to low power. Also, note that no overlap was found in the PFC across species and that the monkey data did not contain BLA data.

**Supplementary Figure S7** Differentially Spliced Associated with AUD Show Minimal Overlap Across Brain Regions

**
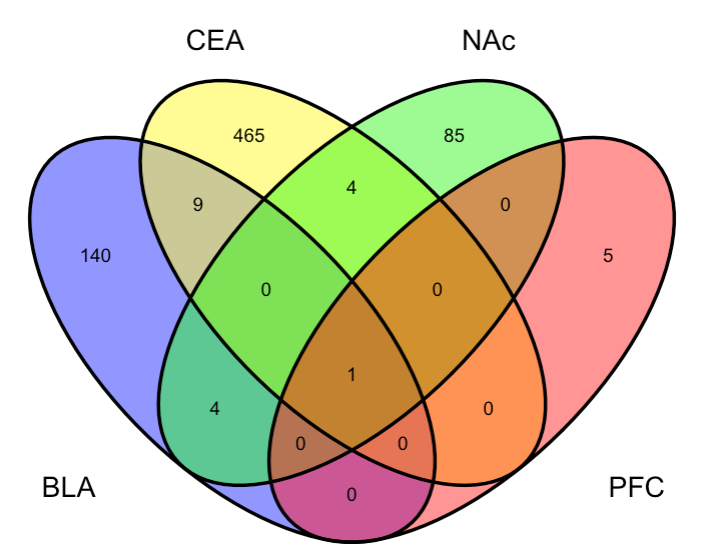
**

Venn diagram shows the 713 genes associated with AUD. The one gene that overlapped across all brain regions was: *RP11-274B21.1.*

**Supplementary Figure S8** Splicing TWAS Associations across Substance Use Traits

Correlation plot showing the linear associations from the 1,397 significant Splicing TWAS associations (FDR < 0.05) across all substance use traits. Note only the correlation between cigarettes per day and opioid use disorder was non significant (p > 0.05).
